# A Clinical Scoring Model to Predict Post‐Endoscopic Retrograde Cholangiopancreatography Pancreatitis in Biliary Endoscopic Retrograde Cholangiopancreatography for Patients With Intact Papilla: A Large Multicenter Prospective Cohort Study

**DOI:** 10.1002/deo2.70245

**Published:** 2025-11-17

**Authors:** Koichi Fujita, Shujiro Yazumi, Hisakazu Matsumoto, Masanori Asada, Hiroko Nebiki, Keiji Hanada, Kazuya Matsumoto, Toru Maruo, Mamoru Takenaka, Hideki Kamada, Chishio Noguchi, Hidefumi Nishikiori, Toshifumi Doi, Teru Kumagi, Takao Iemoto, Nobuaki Azemoto, Takashi Kawamura, Hirofumi Kawamoto, for the Bilio‐pancreatic Study Group of West Japan

**Affiliations:** ^1^ Department of Gastroenterology and Hepatology Yodogawa Christian Hospital Osaka Japan; ^2^ Department of Gastroenterology and Hepatology Medical Research Institute Kitano Hospital PIIF Tazuke Kofukai Osaka Japan; ^3^ Department of Gastroenterology Japanese Red Cross Wakayama Medical Center Wakayama Japan; ^4^ Department of Gastroenterology and Hepatology Japanese Red Cross Osaka Hospital Osaka Japan; ^5^ Department of Gastroenterology Osaka City General Hospital Osaka Japan; ^6^ Department of Gastroenterology JA Onomichi General Hospital Hiroshima Japan; ^7^ Department of Multidisciplinary Internal Medicine Tottori University Faculty of Medicine Tottori Japan; ^8^ Department of Gastroenterology Fukuoka University Chikushi Hospital Fukuoka Japan; ^9^ Department of Gastroenterology and Hepatology Kindai University Osaka Japan; ^10^ Department of Gastroenterology and Neurology Faculty of Medicine Kagawa University Kagawa Japan; ^11^ Department of Gastroenterology Shin Beppu Hospital Oita Japan; ^12^ Department of Gastroenterology Oita San‐ai Medical Center Oita Japan; ^13^ Molecular Gastroenterology and Hepatology Graduate School of Medical Science Kyoto Prefectural University of Medicine Kyoto Japan; ^14^ Department of Gastroenterology and Metabology Ehime University Graduate School of Medicine Ehime Japan; ^15^ Department of Gastroenterology Kita‐Harima Medical Center Hyogo Japan; ^16^ Department of Gastroenterology Shikoku Cancer Center Ehime Japan; ^17^ Kyoto University Health Service Kyoto Japan; ^18^ Department of Internal Medicine Kawasaki Medical School General Medical Center Okayama Japan

**Keywords:** acute pancreatitis, adverse event, clinical prediction rule, endoscopic retrograde cholangiopancreatography, post‐ERCP pancreatitis

## Abstract

**Objectives:**

The risk of post‐endoscopic retrograde cholangiopancreatography (ERCP) pancreatitis (PEP) needs to be predicted in order to take adequate preventive measures in individual cases. The aim of this study was to develop a clinical prediction rule for PEP in biliary ERCP for patients with intact papilla.

**Methods:**

We conducted a multicenter prospective cohort study to investigate the adverse events of biliary ERCP in patients with intact papilla. A total of 3739 patients were prospectively enrolled at 36 hospitals in western Japan between April 2017 and March 2018. Data on patient‐related factors, operator‐related factors, procedure‐related factors, and preventative measures were collected. A multivariable logistic regression was used to identify predictors for PEP, and a scoring model was developed.

**Results:**

The scoring model included 11 factors: age younger than 50 years (2 points), female sex (1 point), ASA 3 (‐2 points), cholangitis (‐1 point), hypoamylasemia (‐1 point), obstruction of the main pancreatic duct at the pancreatic head (‐2 points), cannulation time >20 min (1 point), guidewire insertion into the pancreatic duct (3 points), intraductal ultrasonography (1 point), biopsy (1 point), and total procedure time >60 min (2 points). The area under the receiver operating characteristic curve of this model was 0.729. All cases were stratified into three groups according to the total score: low‐risk, intermediate‐risk, and high‐risk groups, with an incidence of PEP of 1.6%, 6.0%, and 17.7%, respectively.

**Conclusions:**

This scoring model stratified the risk of PEP in individual ERCP cases with intact papilla fairly well.

**Abbreviations**
ASA‐PS: American Society of Anesthesiologists Physical Status
AUC: area under the receiver operating characteristic curve
CI: confidence interval
ERCP: endoscopic retrograde cholangiopancreatography
IDUS: intraductal ultrasonography
MPD: main pancreatic duct
NSAIDs: non‐steroidal anti‐inflammatory drugs
OR: odds ratio
PEP: post‐ERCP pancreatitis
PPS: prophylactic pancreatic stenting
ROC: receiver operating characteristic
SOD: sphincter of Oddi dysfunction

## 1 Introduction

Endoscopic retrograde cholangiopancreatography (ERCP) is an invaluable procedure for the diagnosis and management of pancreaticobiliary diseases; however, reductions in and the management of various adverse events remain crucial issues. Post‐ERCP pancreatitis (PEP) is the most important adverse event of ERCP, and several preventive measures have been proposed. In decision‐making for adequate preventive measures, it is necessary to estimate the risk of PEP in individual cases. Although several prediction models have been reported, most have been from single‐center or small multi‐center retrospective studies with low generalizability [1, 2, 3, 4, 5, 6, 7, 8, 9].

Patient selection is also important for predicting the risk of PEP. Patients with intact papilla are important targets because cannulation is very difficult and the risk of PEP is higher than in post‐sphincterotomy cases [5, 10]. Furthermore, ERCP of the biliary and pancreatic ducts needs to be discussed separately due to differences in the nature of pancreatic procedures. Pancreatic injection may occur accidentally in biliary ERCP, whereas it is intentional and indispensable in pancreatic ERCP. Pancreatic stenting is a prophylactic procedure in biliary ERCP and a therapeutic procedure in pancreatic ERCP. An analysis of ERCP without the distinction of the biliary and pancreatic tracts may lead to inappropriate findings [11]. Therefore, we consider biliary ERCP for patients with intact papilla to be the most clinically important target for the prevention and risk prediction of PEP. The aim of the present study was to develop a reliable prediction rule for PEP in biliary ERCP for patients with intact papilla based on a large multicenter prospective cohort study.

## 2 Material and Methods

### 2.1 Setting/Participants

We conducted a multicenter prospective cohort study that investigated the incidence of adverse events of biliary ERCP in patients with intact papilla. This was a sub‐analysis of a previous cohort study [11]. A total of 3739 patients were prospectively enrolled at 36 hospitals of the Biliopancreatic Study Group of West Japan between April 2017 and March 2018. Patients who underwent ERCP for biliary diseases with intact papilla were extracted for the present study. Among them, patients with comorbid acute pancreatitis, an altered gastrointestinal anatomy, a history of pancreatic surgery, or a severe life‐threatening systemic disease with an American Society of Anesthesiologists Physical Status (ASA‐PS) ≥4 were excluded. In addition, each patient was enrolled only once.

### 2.2 Outcome

The primary outcome was incident PEP. The diagnosis of PEP was based on the consensus definition: new or worsened abdominal pain, new or prolonged hospitalization for at least 2 days, and a serum amylase level measured more than 24 h after the procedure that was ≥3‐fold higher than the upper limit of normal [12, 13, 14, 15]. The severity of PEP was defined by the lexicon for endoscopic adverse events [15].

### 2.3 Predictors

We assessed patient‐related, operator‐related, and procedure‐related factors as candidate predictors. Patient‐related factors included age younger than 50 years, female sex, ASA‐PS 3, previous pancreatitis, suspected sphincter of Oddi dysfunction (SOD), cholangitis, obstruction of the main pancreatic duct (MPD) at the pancreatic head, an abnormal serum amylase level before ERCP (hyperamylasemia and hypoamylasemia), normal serum bilirubin (total bilirubin <1.2 mg/dL), non‐dilated extrahepatic bile ducts (<10 mm), and periampullary diverticulum. Operator‐related factors included a poorly experienced first operator and a low‐volume center. The ERCP operator was judged as poorly experienced if they had performed fewer than 200 procedures or the current number of procedures was <40 per year [16]. A low‐volume center was defined as fewer than 400 ERCPs per year [16]. Procedure‐related factors included precut sphincterotomy, cannulation time >20 min, unsuccessful biliary cannulation, biliary sphincterotomy, endoscopic papillary balloon dilation, endoscopic papillary large balloon dilation, pancreatic injection, guidewire insertion into the pancreatic duct, endoscopic biliary stenting, endoscopic nasobiliary drainage, self‐expandable metallic stent, extraction of biliary stones, brushing cytology, forceps biopsy, intraductal ultrasonography (IDUS), and total procedure time >60 min.

### 2.4 Statistical Analysis

We selected representative predictors in consideration of their multicollinearity. A univariable analysis was then performed using the chi‐squared test for each potential predictor. A multivariable logistic regression with backward stepwise variable selection was used to identify predictors of PEP, and a scoring model was developed based on their β coefficients. To generate a simple integer‐based point score for each predictor variable, scores were given by multiplying the β coefficient by 10 and rounding up or down to the nearest integer. The overall risk score for each patient was calculated by summing the scores of all components.

The performance of the model was evaluated based on calibration, discrimination, and clinical convenience. To assess the calibration of the scoring system, the incidence of PEP was plotted against the total score, and a visual inspection of the histogram and goodness‐of‐fit test was performed. To assess the discrimination of the scoring system, we drew a receiver operating characteristic (ROC) curve for the risk of PEP and calculated the area under the receiver operating characteristic curve (AUC). Overfitting and optimism were evaluated using the bootstrap method by sampling with replacements for 2000 iterations. The area under the ROC curve was calculated in each resampling. Optimism was calculated as the difference between training performance and bootstrap performance [17, 18]. Moreover, the risk stratification of PEP was conducted based on the total scores of individual cases.

Analyses were performed using R 4.2.2 (The R Foundation for Statistical Computing Platform, Vienna, Austria) and JMP18 (SAS Institute, Cary, NC, USA). All tests of significance were two‐tailed, and *p*‐values < 0.05 were considered to be significant.

## 3 Results

A total of 3739 ERCP procedures were registered at the 36 centers. Of the 3739 ERCP procedures, 2362 were performed at 20 general acute hospitals, 1264 at 14 university hospitals, and 113 at two cancer centers. The mean age of study patients was 72.5 (SD 13.1) years, and 1633 were female. The ASA‐PS class was class 1 in 1803 patients, class 2 in 1352, and class 3 in 584. PEP developed in 258 patients (6.9%); 201 mild, 39 moderate, 17 severe, and one fatal. The baseline characteristics, the indications for ERCP, and the incidence of PEP are shown in Table 1. Non‐steroidal anti‐inflammatory drugs (NSAIDs) were administered to 618 patients, of whom 383 (62.0%) were administered NSAIDs before ERCP and 235 (38.0%) immediately after ERCP. The incidence of PEP among patients receiving NSADs was 7.3% and 14.0% respectively. The dosage of NSAIDs was 12.5 mg (*n* = 1), 25 mg (*n* = 253), 50 mg (*n* = 359), 75 mg (*n* = 3), and 100 mg (*n* = 2). A low dose (50 mg or less) was administered to 613 patients (99.2%). Prophylactic pancreatic stenting was performed in 377 patients, and the incidence of PEP in these patients was 11.7% [11].

**TABLE 1:** Baseline characteristics and incidence of post‐endoscopic retrograde cholangiopancreatography pancreatitis (PEP).

Patients, n	3739
Sex, *n* (male/female)	2106/1633
Age, mean, (SD)	72.5, (SD 13.1)
ASA‐PS, *n* (1/2/3)	1803/1352/584
Indications for ERCP, *n*	
Choledocholithiasis	2106
Cholangitis (unknown)	114
Cholecystitis/Mirizzi synd.	21
Bile leakage	41
Benign biliary stricture	85
Sclerosing cholangitis	8
Pancreaticobiliary maljunction	19
Suspected sphincter of Oddi dysfunction	2
Other (non‐neoplastic)	76
Cholangiocarcinoma	408
Gallbladder carcinoma	128
Ampullary neoplasm	62
Hepatocellular carcinoma	17
Pancreatic carcinoma	439
Other (neoplastic)	213
Post‐ERCP pancreatitis, *n* (%)	258 (6.9%)
Severity of PEP; *n* (mild/moderate/severe/fatal)	201/39/17/1

In the univariable analysis, the following factors were significant at *p* < 0.05: age younger than 50 years, female sex, ASA‐PS 3, hyperamylasemia before ERCP (>130 U/L), hypoamylasemia before ERCP (<37 U/L), normal serum bilirubin (total bilirubin <1.2 mg/dL), cholangitis, obstruction of the MPD at the pancreatic head, a poorly experienced first operator, precut sphincterotomy, cannulation time >20 min, pancreatic injection, guidewire insertion into the pancreatic duct, extraction of biliary stones, brushing cytology, biopsy, IDUS, and total procedure time >60 min (Table 2).

**TABLE 2:** Candidate predictors in the univariable analysis.

				Univariable analysis	
Factors	*n*	PEP	PEP (%)	OR (95% CI)	*p*‐Value
Patient‐related factors					
Age younger than 50 years	225	24	10.7	1.67 (1.03–2.63)	0.0305
Female sex	1633	130	8	1.34 (1.03–1.74)	0.0287
ASA‐PS 3	584	24	4.11	0.53 (0.35–0.82)	0.0024
Previous pancreatitis	76	7	9.21	1.38 (0.63–3.03)	0.4225
Suspected sphincter of Oddi dysfunction	34	3	8.82	1.31 (0.40–4.31)	0.6567
Hyperamylasemia before ERCP (>130 U/L)	473	22	4.65	0.62 (0.40–0.97)	0.036
Hypoamylasemia before ERCP (<37 U/L)	500	20	4	0.53 (0.31–0.84)	0.0079
Normal serum bilirubin (Total bilirubin <1.2 mg/dL)	1407	121	8.6	1.51 (1.17–1.95)	0.0014
Nondilated extrahepatic bile ducts (<10 mm)	1976	149	7.54	1.24 (0.96–1.60)	0.0998
Cholangitis	1306	60	4.59	0.54 (0.40–0.73)	< 0.0001
Obstruction of the MPD at the pancreatic head	360	13	3.61	0.48 (0.27–0.85)	0.0096
Periampullary diverticulum	910	51	5.6	0.75 (0.54–1.02)	0.0682
Operator‐related factors					
Poorly experienced first operator	1837	143	7.78	1.3 (1.02–1.69)	0.0361
Low‐volume center (<400 ERCP/year)	738	45	6.1	0.85 (0.61–1.18)	0.3369
Procedure‐related factors					
Wire‐guided cannulation	841	65	7.73	1.17 (0.88–1.57)	0.2815
Precut sphincterotomy	206	24	11.65	1.87 (1.19–2.91)	0.0054
Cannulation attempts duration >20 min	679	88	13	2.53 (1.90–3.35)	< 0.0001
Unsuccessful biliary cannulation	115	12	10.43	1.6 (0.87–2.95)	0.1288
Biliary sphincterotomy	2368	149	6.29	0.78 (0.60–1.00)	0.0531
Endoscopic papillary balloon dilation	145	10	6.9	1 (0.52–1.92)	0.9986
Endoscopic papillary large balloon dilation	122	5	4.1	0.57 (0.23–1.40)	0.2144
Pancreatic injection	1480	154	10.41	2.41 (1.86–3.11)	< 0.0001
Guidewire insertion into the pancreatic duct	1257	158	12.6	3.42 (2.62–4.49)	< 0.0001
Endoscopic biliary stenting	1615	121	7.49	1.17 (0.91–1.51)	0.1978
Endoscopic nasobiliary drainage	719	43	5.98	0.83 (0.59–1.16)	0.278
Self‐expandable metallic stent	221	19	8.6	1.29 (0.79–2.10)	0.2964
Extraction of biliary stones	1269	70	5.52	0.71 (0.53–0.94)	0.0167
Brushing cytology	368	37	10.05	1.59 (1.10–2.30)	0.012
Biopsy	355	41	11.55	1.91 (1.34–2.71)	0.0003
Intraductal ultrasonography	368	43	11.68	1.94 (1.37–2.75)	0.0001
Total procedure time >60 min	509	73	14.34	2.77 (2.07–3.69)	< 0.0001

The following factors were significant in the multivariate analysis; age younger than 50 years (odds ratio [OR]: 1.78 [95% confidence interval [CI], 1.10–2.79]), female sex (OR: 1.33 [95% CI, 1.02–1.73]), ASA 3 (OR: 0.62 [95% CI, 0.39–0.95]), cholangitis (OR: 0.72 [95% CI, 0.52–0.98]), hypoamylasemia (OR: 0.62 [95% CI, 0.37–0.97]), obstruction of the MPD at the pancreatic head (OR: 0.41 [95% CI, 0.22–0.71]), cannulation time >20 min (OR: 1.43 [95% CI, 1.03–1.97]), guidewire insertion into the pancreatic duct (OR: 2.88 [95% CI, 2.17–3.82]), IDUS (OR: 1.49 [95% CI, 1.00–2.17]), biopsy (OR: 1.51 [95% CI, 1.00–2.23]), and total procedure time >60 min (OR:1.64 [95% CI, 1.17–2.28]).

### 3.1 Construction of the Scoring System

The probability of PEP was calculated using the following equation:

1/ (1+e^−2.58^×e^‐[0.29 × age younger than 50 years old + 0.14 × female – 0.24 × ASA 3 – 0.16 ×^ ^cholangitis – 0.24 × hypoamylasemia – 0.45 × obstruction of the MPD at the pancreatic head +^ ^0.21 × cannulation time >20 min + 0.53 × guidewire insertion into the pancreatic duct + 0.20^ ^× IDUS + 0.21 × biopsy + 0.24 × total procedure time >60 min]^)

≈ 1/ (1+e^−2.58^ × e^−0.15 [2 × age younger than 50 years old + 1 × female – 2 × ASA 3 – 1^ ^× cholangitis – 2 × hypoamylasemia – 3 × obstruction of the MPD at the pancreatic head + 1^ ^× cannulation time >20 min + 3 × guidewire insertion into pancreatic duct + 1 × IDUS + 1^ ^× biopsy + 2 × total procedure time >60 min]^) = 1/ (1+e^−2.58^×e^−0.15 × total score^)

A scoring system was constructed from six patient‐related and five procedure‐related factors as follows: age younger than 50 years: 2 points; female sex: 1 point; ASA 3: ‐2 points; cholangitis: ‐1 point; hypoamylasemia: ‐1 point; obstruction of the MPD at the pancreatic head: ‐2 points; cannulation time >20 min: 1 point; guidewire insertion into the pancreatic duct: 3 points; IDUS: 1 point; biopsy: 1 point; and total procedure time >60 min: 2 points (Table 3). Points were summed to a total score that predicted the risk of pancreatitis.

**TABLE 3:** Predictors in the multivariable analysis and their scores.

Predictors	β	OR (95%CI)	*p*‐Value	Score
Patient‐related factors				
Age younger than 50 years	0.293	1.78 (1.10–2.79)	0.0141	**2**
Female sex	0.141	1.33 (1.02–1.73)	0.0323	**1**
ASA 3	−0.238	0.62 (0.39–0.95)	0.0345	**−2**
Cholangitis	−0.163	0.72 (0.52–0.98)	0.0401	**−1**
Hypoamylasemia (<37 U/L)	−0.244	0.62 (0.37–0.97)	0.0478	**−2**
Obstruction of the MPD at the pancreatic head	−0.451	0.41 (0.22–0.71)	0.0028	**−3**
Procedure‐related factors				
Cannulation time >20 min	0.206	1.43 (1.03–1.97)	0.031	**1**
Guidewire insertion into the pancreatic duct	0.526	2.88 (2.17–3.82)	<0.0001	**3**
Intraductal ultrasonography	0.199	1.49 (1.00–2.17)	0.0438	**1**
Biopsy	0.208	1.51 (1.00–2.23)	0.0442	**1**
Total procedure time >60 min	0.237	1.64 (1.17–‐2.28)	0.0037	**2**

### 3.2 Performance of the Scoring System

**FIGURE 1:**
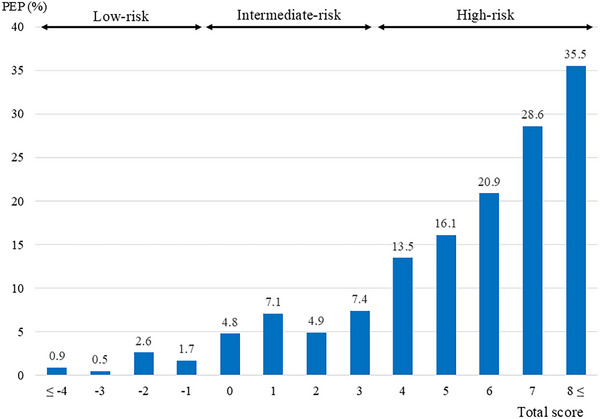
Incidence of post‐endoscopic retrograde cholangiopancreatography (ERCP) pancreatitis according to the total score and risk stratification.

The actual incidence of PEP increased as the total score became higher (Figure 1). The p‐value of the goodness‐of‐fit test was 0.333, indicating an acceptable fit. The AUC of this scoring model was 0.729. A bootstrap analysis (i.e., resampling the model 2000 times) revealed a mean over the optimism value of 0.012 (95% CI: ‐0.019–0.041) and corrected AUC of 0.716.

### 3.3 Risk Stratification of PEP

All cases were divided into three groups according to the total score: low‐risk (scoring ‐1 point or less), intermediate‐risk (between 0 and 3 points), and high‐risk (more than 4 points). The incidence of PEP was 1.6% in the low‐risk group (17/1048), 6.0% in the intermediate‐risk group (121/2012), and 17.7% in the high‐risk group (120/679) (Table 4).

**TABLE 4:** Risk stratification by scores.

Risk group	Scoring	*n*	PEP, *n* (%)	Severity of PEP
				mild/moderate/severe/fatal
Low‐risk	≤‐1 point	1048	17 (1.6 %)	15/1/1/0
Intermediate‐risk	0–3 points	2012	121 (6.0 %)	96/18/7/0
High‐risk	≥4 points	679	120 (17.7 %)	90/20/9/1

## 4 Discussion

This prediction model, consisting of 11 factors, showed good discrimination and risk stratification. It is highly practical because it is limited to biliary ERCP for patients with intact papilla and consists of clinically available predictors. Furthermore, this model is highly reliable because it is derived from a prospective large‐scale multicenter study using clearly defined and well‐planned data collection.

We propose a two‐step score calculation and risk prediction. In the first step, a physician predicts the risk of PEP before ERCP based on patient‐related factors and planned procedures, such as IDUS and/or biopsy. In the second step, during and at the end of ERCP, the physician adds up the scores for the cannulation time, guidewire insertion into the pancreatic duct, and total procedure time because these factors become known during ERCP procedures. Based on the risk prediction, the physician considers the necessity of additional preventive measures and also the intensity of patient management after ERCP.

We propose three preventive measures for high‐risk patients. The first measure involves the rectal administration of high‐dose NSAIDs. NSAIDs are recommended as the main preventive measure and are administered before ERCP in many cases, but need to be added immediately after ERCP for high‐risk patients who did not receive them before ERCP [19, 20]. In this study, rectal administration of low‐dose NSAIDs before ERCP did not show any preventive effect, whereas the incidence of PEP was high in patients who were administered low‐dose NSAIDs after ERCP. [11] This suggests that post‐ERCP NSAIDs reflected various factors related to the operator's choice, such as the actual difficulty of the procedures and the patient's conditions. The second measure is aggressive hydration for patients without contraindications to high‐volume hydration [20, 21, 22, 23, 24]. The last measure is prophylactic pancreatic stenting (PPS) for patients who have undergone guidewire insertion into the pancreatic duct [14, 25, 26]. Pancreatic stenting can be performed as an additional preventive measure at the end of ERCP in such high‐risk patients. In contrast, we propose omitting additional preventive measures for low‐risk patients.

Two prediction models using machine learning were recently reported [8, 9]. Machine learning may be used to improve prediction accuracy; however, it is complex and difficult to understand clinically. On the other hand, our model is clinically interpretable and easy to use at the bedside because of its simple structure, using only clinically important factors. However, integrating this model into an electronic risk calculator in the future could potentially further enhance its performance and generalizability.

Among the patient‐related factors included in this model, age younger than 50 years and female sex are well‐known risk factors for PEP. ASA‐PS3 may reflect patient backgrounds, such as old age, and cholangitis is a factor that facilitates bile duct cannulation because of high intrabiliary pressure and viscous bile. The protective factors, hypoamylasemia and obstruction of the MPD at the pancreatic head, are considered to be associated with impaired pancreatic exocrine function. All procedure‐related factors in this model are well‐known risk factors [20, 27]. Various cut‐offs have been proposed for the cannulation time; however, in this model, 20 min was a useful cut‐off point for predicting the risk of PEP.

This study has several limitations that need to be addressed. External validity was not assessed because this model was developed using a full dataset to improve predictive accuracy and clinical validity. However, we consider our model to have high generalizability because it is based on prospectively collected data from a large number of hospitals. We hope that other researchers will evaluate the external validity of this model in different settings. Moreover, the AUC of this model was 0.729, which was not very high, indicating moderate discrimination. This is because the present study targeted patients with intact papilla, a relatively high‐risk population. However, risk stratification for PEP is fully feasible and clinically useful. Furthermore, this model did not include preventive measures. The rectal administration of NSAIDs, most of which were at low doses of 50 mg or less, did not exert preventive effects in this cohort [11]. Aggressive intravenous infusion, a candidate preventive measure for PEP, was not performed because evidence was not established at the time of this study. Moreover, we did not consider including preventive measures, such as PPS, in the prediction model because they are affected by the subjective judgment of endoscopists. We also considered it preferable not to include preventive measures in the predictive model in order to identify preventive measures after the risk assessment. However, future development and evaluation of PEP prevention strategies incorporating this scoring model are needed.

In conclusion, this scoring system may be useful for easily predicting and stratifying the risk of PEP in individual cases in clinical practice. Aggressive preventive measures are recommended for patients in the high‐risk group with a score ≥4. Future external validation and integration into electronic risk calculators may enhance generalizability and clinical utility.

## Author Contributions

KF, SY, KM, TM, MT, TKw, and HKw conceived the original concept and design of the study. KF, SY, HM, MA, HNb, KH, KM, TM, MT, HKm, CN, HNs, TD, TKm, TI, NA, TKw, and HKw were involved in data collection and sample management. KF, SY, and TKw performed data analysis and interpretation. KF, SY, and TKw drafted the work. All authors critically revised this article and approved the final version for publication.

## Conflicts of Interest

The authors declare no conflicts of interest for this article. Hirofumi Kawamoto has received consulting fees from Gadelius, Piolax, Kaneka, and Japan Life Line, but these have not influenced the content or conclusions of this study.

## Funding

The authors received no specific funding for this work.

## Ethics Statement

**Approval of the research protocol by an Institutional Reviewer Board**: This study was approved by the Institutional Review Boards of the respective institutions.

## Consent

This was a non‐invasive observational study, and we did not collect biological samples for research purposes. We informed the subjects of the outline of this study on the website or bulletin board of each hospital, and provided an opportunity to decline participation.

## Clinical Trial Registration

This study was registered in the University Hospital Medical Information Network (UMIN000024820).
